# Clinical characteristics and prognosis analysis of secondary intestinal diffuse large B-cell lymphoma: a multi-center retrospective study over a decade

**DOI:** 10.3389/fonc.2026.1905087

**Published:** 2026-10-27

**Authors:** Xiaojun Chen, Zhimin Yan, Jianzhen Shen, Donghui Gan, Minjuan Zeng, Yue Yin, Zuotao Li, Hehui Zhang, Yanquan Liu

**Affiliations:** 1 Department of Hematology, The Affiliated Hospital of Putian University, Putian, Fujian, China; 2 Department of Hematology, The First Affiliated Hospital of Gannan Medical University, Ganzhou, Jiangxi, China; 3 Fujian Institute of Hematology, Fujian Medical University Union Hospital, Fuzhou, Fujian, China; 4 The School of Basic Medical Sciences, Guangdong Medical University, The Affiliated Hospital of Guangdong Medical University, Zhanjiang, Guangdong, China; 5 Department of Hematology, Ganzhou People’s Hospital, The Affiliated Ganzhou Hospital, Jiangxi Medical College, Nanchang University, Ganzhou, Jiangxi, China

**Keywords:** clinical characteristics, diffuse large B-cell lymphoma, malignant hematological tumors, prognostic factors, secondary tumor

## Abstract

**Background and objective:**

Secondary intestinal diffuse large B-cell lymphoma (SI-DLBCL) is a clinically rare hematological malignancy characterized by high invasiveness and heterogeneity. Due to its nonspecific clinical manifestations, it is often misdiagnosed or overlooked in clinical practice, leading to poor patient outcomes. This study aims to systematically explore and analyze the clinical characteristics and prognostic factors of SI-DLBCL, providing valuable insights for basic research and clinical management of secondary lymphomas at rare sites within the field of Hematology.

**Methods:**

This multicenter retrospective study enrolled 483 patients diagnosed with SI-DLBCL between January 1, 2015, and December 31, 2025, spanning an 11-year period; clinical data were retrospectively collected from five tertiary medical centers across different regions of China: Fujian Medical University Union Hospital, Affiliated Hospital of Putian University, the First Affiliated Hospital of Gannan Medical University, Affiliated Hospital of Guangdong Medical University and the Affiliated Ganzhou Hospital of Nanchang University. The data included clinical and pathological features, treatment regimens, and prognosis. Univariate and multivariate Cox regression analyses were conducted to identify significant prognostic factors.

**Results:**

Among the 483 patients with SI-DLBCL, 298 cases were male (61.70%) and 185 cases were female (38.30%), with a median age of onset of 60.5 years (range: 18–84 years). Clinical manifestations were predominantly nonspecific, and the first-line treatment primarily consisted of chemotherapy. The median follow-up duration was 70 months (range: 0.5–135 months). Specifically, 322 cases (66.67%) received chemotherapy alone, 146 cases (30.23%) received a combination of chemotherapy and surgical intervention, and 15 cases (3.10%) underwent surgery alone. The overall response rate (ORR), defined as the sum of CR and PR, shows significant differences between the three groups for patients with SI-DLBCL (*P* = 0.002). Age, Lugano stage, IPI score, serum LDH, albumin (ALB), β2 microglobulin, first-line treatment showed statistically significant differences between the survival group and the death group (*P* < 0.05). Univariate Cox regression analysis identified age, Lugano stage, IPI score, LDH, ALB, β2-microglobulin, and first-line treatment as significant predictors of OS and PFS. Multivariate analysis confirmed that age and Lugano stage were independent prognostic factors affecting both OS and PFS. The results of univariate logistic analysis showed that age, Lugano stage, IPI score, serum LDH, ALB, β2 microglobulin, first-line treatment were risk factors for mortality in SI-DLBCL patients (all *P* < 0.05). Multivariate logistic regression analysis confirmed that advanced age and advanced Lugano stage served as independent risk factors for all-cause mortality. In contrast, patients aged ≤60 years, with Lugano stage I–II disease, IPI scores of 0–2, normal serum LDH levels, serum albumin ≥35 g/L, β2-microglobulin ≤3 μg/ml, and those receiving combined chemotherapy plus surgical resection exhibited significantly more favorable long-term prognosis in univariate analysis.

**Conclusion:**

SI-DLBCL predominantly affects middle-aged and elderly men, with nonspecific early clinical manifestations. Chemotherapy, particularly the R-CHOP regimen, remains the primary treatment modality. For patients with localized intestinal lesions or acute intestinal complications (perforation, massive bleeding, or obstruction), surgical intervention combined with rituximab-based systemic chemotherapy may be considered as a multidisciplinary treatment strategy; however, due to the retrospective study design and confounding by indication, this observation should be further validated in prospective studies. Age and Lugano stage are independent prognostic factors influencing OS and PFS in patients with SI-DLBCL.

## Introduction

Non-Hodgkin’s lymphoma (NHL) is a malignant proliferative tumor originating from immune cells. Its classification is complex and highly heterogeneous, with more than 50 subtypes listed in the latest World Health Organization (WHO) classification. Diffuse large B-cell lymphoma (DLBCL) is the most common pathological subtype of NHL, characterized by high heterogeneity and invasiveness. Gastrointestinal lymphoma accounts for approximately 30% to 40% of all extranodal NHL cases, with the stomach being the most frequent site of onset, followed by the intestine (1, 2). Secondary intestinal diffuse large B-cell lymphoma (SI-DLBCL) is a rare hematological malignancy in clinical practice. However, its incidence and mortality rates in adults have been increasing recently, drawing significant attention.

Regrettably, the pathogenesis of SI-DLBCL remains unclear at present. Patients with SI-DLBCL are predominantly middle-aged and elderly men. Early clinical manifestations lack specificity and may include non-specific symptoms such as abdominal pain, low back pain, fatigue, fever, vomiting, and weight loss (3, 4). Clinically, distinguishing SI-DLBCL from other intestinal malignancies is challenging (5, 6). The gold standard for diagnosing SI-DLBCL is histopathological biopsy, while the first-line treatment primarily involves chemotherapy (7, 8). Additional therapeutic options include radiotherapy, targeted therapy, or surgery (9, 10). Currently, research on SI-DLBCL is limited, with most studies being single-center investigations or case reports. Systematic multi-center integrated data analysis and prospective studies are lacking, resulting in insufficient evidence to support guideline recommendations. Therefore, this study aims to integrate multi-center clinical data, conduct comprehensive data analysis, systematically investigate and clarify the clinical characteristics of SI-DLBCL, evaluate the advantages and disadvantages of diagnostic and treatment strategies, identify prognostic risk factors, and assess the applicability of current treatment protocols. Furthermore, reviewing the latest literature in the field of hematological malignancies is expected to provide a robust scientific basis for improving early diagnosis rates, optimizing treatment decisions, and enhancing long-term prognosis.

## Materials and methods

### Research population

A total of 483 patients with SI-DLBCL admitted to Fujian Medical University Union Hospital, Affiliated Hospital of Putian University, the First Affiliated Hospital of Gannan Medical University, Affiliated Hospital of Guangdong Medical University and the Affiliated Ganzhou Hospital of Nanchang University between January 1, 2015, and December 31, 2025, were selected as research subjects. The technical roadmap of this study is detailed in **Figure 1**. All patients underwent complete treatment courses (diagnosis, treatment, and rehabilitation) within these hospitals, ensuring comprehensive data collection. Ethical approval was obtained from the medical ethics committee of each center, adhering to the Declaration of Helsinki. A waiver of informed consent was granted by each institutional ethics committee for this retrospective study using de-identified clinical data, and the study protocol received official ethical clearance.

**Figure 1:**
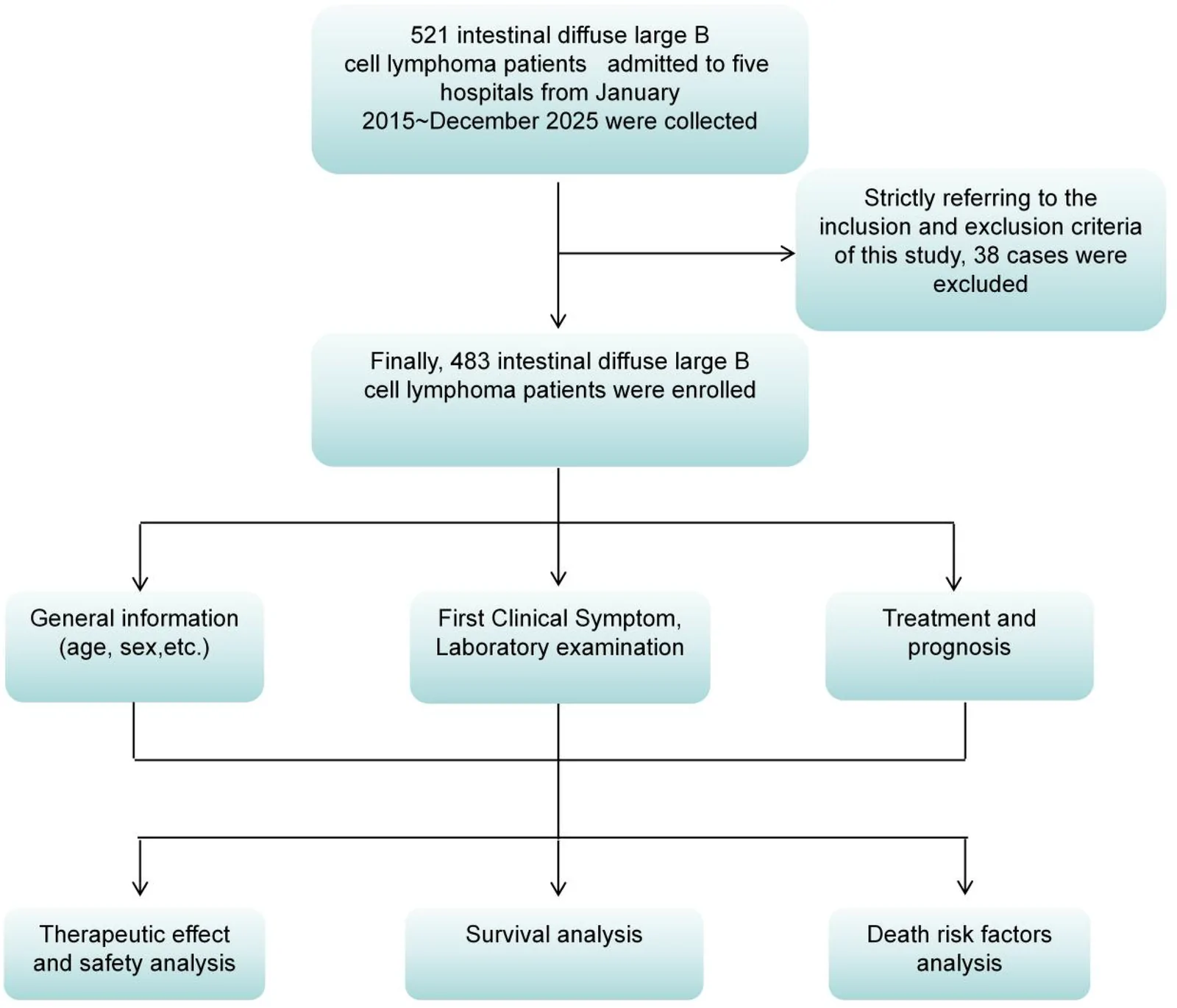
Flow chart of study design and screening sources.

### Diagnostic criteria

Intestinal lymphoma can be categorized into primary and secondary groups. Among the selected patients, those meeting the Dawson diagnostic criteria (11) were classified into the primary group, while those not meeting these criteria were diagnosed with SI-DLBCL. Specifically, in addition to lesions located in the intestine, lymphoma lesions were also present in other sites, with the first diagnosis and onset site not being the intestine. Clinical features, pathological and molecular biological test results were consistent with DLBCL.

Dawson’s diagnostic criteria for primary intestinal DLBCL (PI-DLBCL) are defined as follows: (1) No palpable superficial lymphadenopathy on physical examination (rather than only radiologically unobserved lesions); (2) Absence of mediastinal lymphadenopathy on chest imaging; (3) Normal peripheral white blood cell counts; (4) Disease limited exclusively to the gastrointestinal tract and regional mesenteric lymph nodes, without distant extraintestinal nodal infiltration; (5) No radiological or pathological evidence of hepatic or splenic involvement, including no direct tumor extension from intestinal primary lesions to the liver or spleen. Patients with mild neutrophilia but satisfying all remaining Dawson criteria were still classified as PI-DLBCL.

### Inclusion and exclusion criteria

Inclusion Criteria: (1) Patients with a clear pathological biopsy diagnosis of DLBCL; (2) Intestinal involvement confirmed via endoscopy and surgery, with the lesion site being the intestine; (3) Complete baseline assessment and clinical data available after systematic clinical evaluation, confirming secondary intestinal lymphoma; (4) Patients aware of the study purpose and voluntarily participating; (5) Study content compliant with ethics committee requirements.

Exclusion Criteria: (1) Meeting diagnostic criteria for primary intestinal diffuse large B-cell lymphoma; (2) Patients with incomplete clinical data despite a clear diagnosis; (3) Patients with DLBCL transformed from follicular lymphoma; (4) Patients who received anti-tumor treatment prior to enrollment; (5) Patients with coexisting uncured tumors; (6) Patients with incomplete medical history, non-standard diagnosis and treatment, or those discontinuing treatment midway; (7) Patients refusing inclusion due to privacy concerns or other reasons. Notably, the exclusion of PI-DLBCL does not exclude early-stage (I–II) SI-DLBCL. PI-DLBCL is defined by Dawson criteria as intestinal-origin lymphoma without systemic extraintestinal lesions, which exhibits distinct clinical behavior and treatment response and was therefore excluded to eliminate cohort heterogeneity. In contrast, early-stage SI-DLBCL originates from systemic nodal disease with secondary intestinal infiltration, even when localized to the bowel at diagnosis; these patients share consistent prognostic and therapeutic features with advanced SI-DLBCL and were retained for analysis.

### Collection and organization of clinical data

General demographic characteristics of patients from multiple medical centers were collected and organized, including age at initial diagnosis, gender, ECOG score, Lugano stage, B symptoms, lymphoma location, pathological subtype, IPI score, laboratory test results (white blood cells, serum lactate dehydrogenase, albumin, β2 microglobulin, Ki-67), and first-line treatment methods.

All first-line treatment decisions for enrolled SI-DLBCL patients were formulated in accordance with the 2021 Lugano Classification Guidelines for Lymphoma, the National Comprehensive Cancer Network (NCCN) DLBCL Clinical Practice Guidelines (2015–2025 editions), and the Chinese Guidelines for the Diagnosis and Treatment of Diffuse Large B-Cell Lymphoma (2022). Treatment modality selection (chemotherapy alone vs chemotherapy combined with surgery) followed standardized indications: upfront surgical resection was reserved for patients with intestinal perforation, massive bleeding, complete bowel obstruction, or bulky localized intestinal masses; rituximab-based immunochemotherapy was the backbone systemic therapy for all non-emergency cases.

### Follow-up

Follow-up data were obtained and recorded through outpatient and inpatient medical records and telephone follow-ups. Observation began at the time of diagnosis, with endpoints being patient death or the end of follow-up. Median survival was calculated in months. Follow-up cut-off date: March 31, 2026, or patient death. Median follow-up time was 70 months (range: 0.5–135 months). After applying all inclusion and exclusion criteria (including exclusion of 38 patients with incomplete data or unavailable follow-up), a total of 483 patients were finally enrolled in this study. All 483 patients had complete follow-up data: 116 cases died and 367 cases survived at the time of analysis.

### Statistical analysis

Clinical data were organized using Excel tables and analyzed statistically using IBM SPSS 26.0 software. Normally distributed measurement data were expressed as *Mean* ± *SD*, with t-tests used for comparisons. Non-normally distributed data were expressed as median (interquartile range), with Mann-Whitney tests applied. Count data were expressed as cases (%). Chi-square tests were used for categorical data comparisons. Survival analysis was performed using the Kaplan-Meier method, with Log-rank tests applied. Univariate and multivariate analyses of risk factors for overall survival (OS) and progression-free survival (PFS) were conducted using Cox regression models. For both OS and PFS, variables with P less than 0.10 in the univariate analysis were entered into the multivariate Cox regression model using the forward stepwise (likelihood ratio) method. The proportional hazards assumption for the Cox regression models was verified using Schoenfeld residuals; all covariates met the proportional hazards assumption (all P greater than 0.05). Survival curves were plotted using GraphPad Prism 9.0 software, and remaining clinical data and characteristics were analyzed descriptively. *P* value < 0.05 was considered statistically significant. To account for potential center-level clustering effects in this multi-center study, mixed-effects Cox regression models with center as a random intercept were additionally performed as a sensitivity analysis. The intraclass correlation coefficient (ICC) was calculated to quantify the proportion of variance attributable to between-center differences. All statistical analyses were performed using IBM SPSS 26.0 and R software (version 4.3.0, package ‘coxme’). P less than 0.05 was considered statistically significant.

## Results

### Clinical characteristics

According to the inclusion and exclusion criteria, combined with follow-up data, a total of 483 patients with SI-DLBCL were included in this study. The median follow-up duration was 70 months (range: 0.5–135 months). Among these patients, 367 cases (75.98%) survived, 116 cases (24.02%) died. The 1-year, 3-year, and 5-year progression-free survival (PFS) rates were 75.36%, 50.72%, and 32.92%, respectively, while the 1-year, 3-year, and 5-year overall survival (OS) rates were 78.47%, 56.11%, and 34.37%, respectively. There were 298 male cases (61.70%) and 185 female cases (38.30%), yielding a male-to-female ratio of 1.61:1. The median age at onset was 60.5 years (range: 18–84 years). According to the Lugano staging system, the majority of patients presented with advanced-stage disease (stage III–IV), with 172 cases (35.61%) in stage I–II and 311 cases (64.39%) in stage III–IV. Clinical manifestations were nonspecific and lacked diagnostic specificity. Only 182 cases (37.68%) presented with abdominal pain as their primary complaint. The general baseline data and clinical characteristics of the SI-DLBCL patients included in this study are detailed in **Table 1**.

**Table 1:** Baseline data and clinical characteristics of 483 SI-DLBCL patients.

Clinical characteristics	Results
Age [> 60 year-old, *n* (%)]	224 (46.38)
Gender [female, *n* (%)]	185 (38.30)
ECOG score [≥ 2, *n* (%)]	187 (38.72)
Lugano stage [Stage III-IV, *n* (%)]	311 (64.39)
B symptoms [No, *n* (%)]	324 (67.08)
Hans classification [Non-GCB type, *n* (%)]	279 (57.76)
IPI score [3-5, *n* (%)]	254 (52.59)
WBC	6.50 [5.00, 8.46]
Serum LDH	237 [174, 372]
Albumin	37.80 [34.00,42.30]
β2 Microglobulin	2.63 [2.14,3.80]
Ki-67 (%) [≥ 80%, *n* (%)]	358 (74.12)
Lymphoma site	
Colorectal	204 (42.24%)
Non-colorectal	279 (57.76%)
First-line treatment	
Chemotherapy alone	322 (66.67)
Chemotherapy + surgery	146 (30.23)
Surgery alone	15 (3.10)

### Treatment regimens

The first-line treatment predominantly consisted of chemotherapy. Specifically, 322 cases (66.67%) received chemotherapy alone, 146 cases (30.23%) received a combination of chemotherapy and surgical intervention, and 15 cases (3.10%) underwent surgery alone. Among those receiving chemotherapy alone, the most common regimen was R-CHOP (rituximab, cyclophosphamide, doxorubicin, vincristine, prednisone), administered in 135 cases (41.93%). Other regimens included CHOP (*n* = 20, 6.21%), R2-CHOP (*n* = 58, 18.01%), R-DAEPOCH (*n* = 41, 12.73%), and R-miniCHOP (*n* = 18, 5.59%). Additional regimens such as IR (*n* = 13, 4.04%), chidamide + R-CHOP (*n* = 14, 4.35%), R-COP (*n* = 9, 2.80%), R-CVP (*n* = 5, 1.55%), gemcitabine + CVP (*n* = 5, 1.55%), and zanubrutinib (ZB) + temozolomide (TMZ) + R-reduced-dose MA (*n* = 4, 1.24%) were also utilized. The details of the treatment regimens of SI-DLBCL patients included in this study are depicted in **Figure 2**.

**Figure 2:**
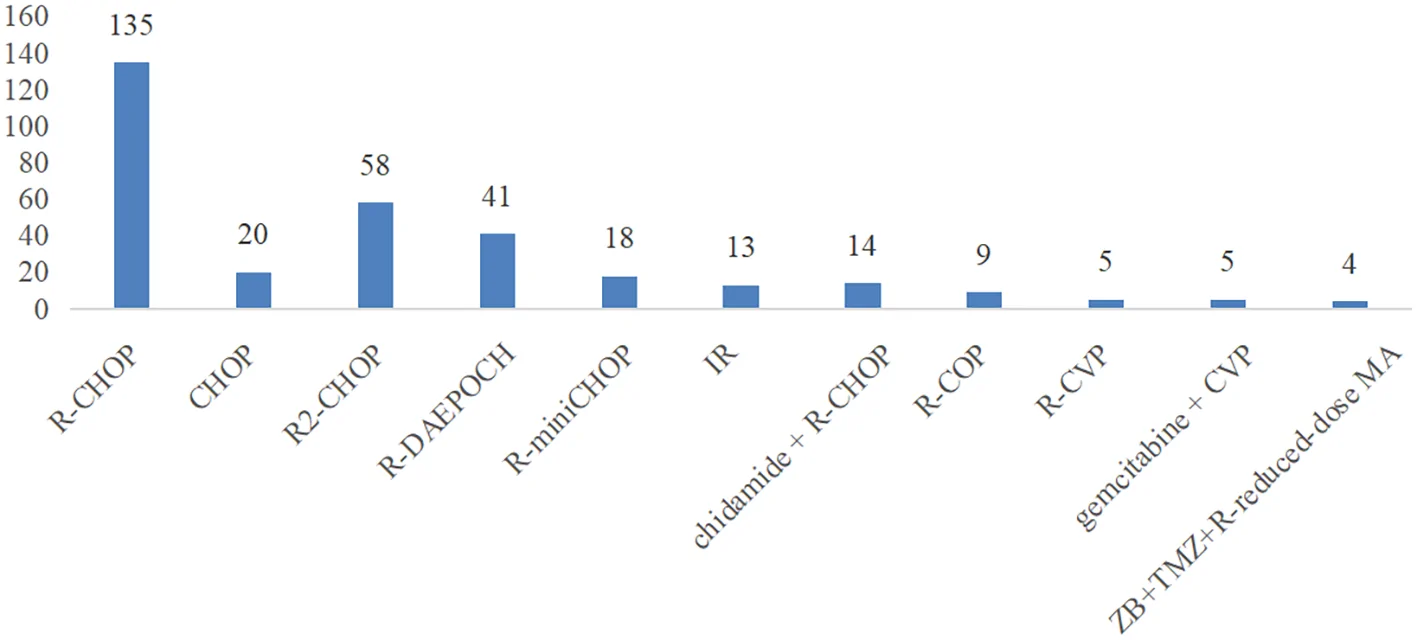
Chemotherapy regimen of 322 patients who received chemotherapy alone.

### Therapeutic effect and safety analysis

As shown in **Table 2**, all patients with SI-DLBCL in our study were divided into three groups based on their first-line treatment modalities: chemotherapy alone, chemotherapy + surgery and surgery alone. The therapeutic effect was evaluated after at least two cycles of treatment using the 2014 Lugano response criteria. The evaluation criteria included complete remission (CR), partial remission (PR), stable disease (SD), and progressive disease (PD). The overall response rate (ORR), defined as the sum of CR and PR, shows significant differences between the three groups for patients with SI-DLBCL (*P* = 0.002). It is noteworthy that the primary adverse reactions observed during the treatment process of SI-DLBCL patients included in this study were granulocytopenia, anemia, thrombocytopenia, hepatic and renal function impairment, gastrointestinal disturbances, neurological damage, and dermatological damage. The results indicated that there were no statistically significant differences in the incidence of these adverse reactions among the three groups (all *P* > 0.05).

**Table 2:** Clinical efficacy and safety analysis of different treatment modalities in patients with SI-DLBCL.

Features	All patients (*n* = 483)	Chemotherapy alone (*n* = 322)	Chemotherapy + surgery (*n* = 146)	Surgery alone (*n* = 15)	*χ*^2^ value	*P* value
Efficacy					12.989	**0.002**
CR+PR	379 (78.47%)	248 (77.02%)	124 (84.93%)	7 (46.67%)		
SD+PD	104 (21.53%)	74 (22.98%)	22 (15.07%)	8 (53.33%)		
Relapsed or refractory to treatment					1.108	0.575
Yes	89 (18.43%)	56 (17.39%)	29 (19.86%)	4 (26.67%)		
No	394 (81.57%)	266 (82.61%)	117 (80.14%)	11 (73.33%)		
Granulocytopenia	243 (50.31%)	165 (51.24%)	78 (53.42%)	0 (0.00%)	–	–
Anemia	154 (31.88%)	102 (31.68%)	47 (32.19%)	5 (33.33%)	0.014	0.993
Thrombocytopenia	183 (37.89%)	127 (39.44%)	55 (37.67%)	1 (6.67%)	3.766	0.152
Hepatic and renal function impairment	112 (23.19%)	74 (22.98%)	36 (24.66%)	2 (13.33%)	0.668	0.716
Gastrointestinal disturbances	304 (62.94%)	201 (62.42%)	109 (74.66%)	10 (66.67%)	1.331	0.514
Neurological damage	138 (28.57%)	97 (30.12%)	41 (28.08%)	0 (0.00%)	–	–
Dermatological damage	53 (10.97%)	37 (11.49%)	16 (10.96%)	0 (0.00%)	–	–

Statistically significant values of test statistics and P values are marked in bold black font, namely P < 0.05.

### Prognostic analysis

As presented in **Table 3**, the general clinical data of 483 patients diagnosed with SI-DLBCL were statistically analyzed. Variables including age, Lugano stage, IPI score, serum LDH level, albumin level, β2 microglobulin level, and first-line treatment regimen demonstrated statistically significant differences between the survival group and the mortality group (all *P* < 0.05). To investigate the impact of various factors on the survival period of 483 patients with SI-DLBCL, a log-rank test was performed for each index. The results indicated that age, Lugano stage, and International Prognostic Index (IPI) score influenced OS, while age impacted PFS. Detailed survival curves for age, Lugano stage, IPI, lactate dehydrogenase (LDH), albumin, β2-microglobulin, and first-line treatment are provided in **Figure 3**. According to the survival curve analysis, patients aged ≤ 60 years, with Lugano stage I–II, IPI score of 0–2, normal LDH levels, albumin ≥ 35 g/L, β2 microglobulin ≤ 3 μg/ml, and those who received a combination of chemotherapy and surgery exhibited a more favorable prognosis (all *P* < 0.05).

**Table 3:** Clinical features and prognostic analysis of SI-DLBCL.

Observed metrics	All patients (*n* = 483)	Survivors (*n* = 367)	Non-survivors (*n* = 116)	*χ*^2^ value	*P* value
Age (year)				24.561	< 0.001
≤ 60	259 (53.62%)	220 (59.95%)	39 (33.62%)		
> 60	224 (46.38%)	147 (40.05%)	77 (66.38%)		
Gender				3.526	0.060
Male	298 (61.70%)	235 (64.03%)	63 (54.31%)		
Female	185 (38.30%)	132 (35.97%)	53 (45.69%)		
ECOG score				0.040	0.842
< 2 points	296 (61.28%)	224 (61.04%)	72 (62.07%)		
≥ 2 points	187 (38.72%)	143 (38.96%)	44 (37.93%)		
Lugano stage				42.503	< 0.001
Stage I-II	172 (35.61%)	160 (43.60%)	12 (10.34%)		
Stage III-IV	311 (64.39%)	207 (56.40%)	104 (89.66%)		
B symptoms				0.002	0.966
Yes	159 (32.92%)	121 (32.97%)	38 (32.76%)		
No	324 (67.08%)	246 (67.03%)	78 (67.24%)		
Hans pathological classification				1.671	0.196
GCB type	204 (42.24%)	161 (43.87%)	43 (37.07%)		
NonGCB type	279 (57.76%)	206 (56.13%)	73 (62.93%)		
IPI score				33.167	< 0.001
0-2	229 (47.41%)	201 (54.77%)	28 (24.14%)		
3-5	254 (52.59%)	166 (45.23%)	88 (75.86%)		
WBC				0.123	0.726
Normal	364 (75.36%)	278 (75.75%)	86 (74.14%)		
Abnormal	119 (24.64%)	89 (24.25%)	30 (25.86%)		
Serum LDH				5.334	0.021
Normal	245 (50.72%)	197 (53.68%)	48 (41.38%)		
Elevated	238 (49.28%)	170 (46.32%)	68 (58.62%)		
Albumin				7.330	0.007
< 35 g/L	147 (30.43%)	100 (27.25%)	47 (40.52%)		
≥ 35 g/L	336 (69.57%)	267 (72.75%)	69 (59.48%)		
β2 Microglobulin				9.790	0.002
≤ 3 μg/ml	285 (59.01%)	231 (62.94%)	54 (46.55%)		
> 3 μg/ml	198 (40.99%)	136 (37.06%)	62 (53.45%)		
Ki-67 (%)				0.062	0.804
< 80%	125 (25.88%)	96 (26.16%)	29 (25.00%)		
≥ 80%	358 (74.12%)	271 (73.84%)	87 (75.00%)		
Lymphoma site				1.678	0.195
Colorectal	204 (42.24%)	149 (40.60%)	55 (47.41%)		
Non-colorectal	279 (57.76%)	218 (59.40%)	61 (52.59%)		
First-line treatment				6.589	0.037
Chemotherapy alone	322 (66.67%)	234 (63.76%)	88 (75.86%)		
Chemotherapy + surgery	146 (30.23%)	122 (33.24%)	24 (20.69%)		
Surgery alone	15 (3.10%)	11 (3.00%)	4 (3.45%)		

Statistically significant values of test statistics and P values are marked in bold black font, namely P < 0.05.

**Figure 3:**
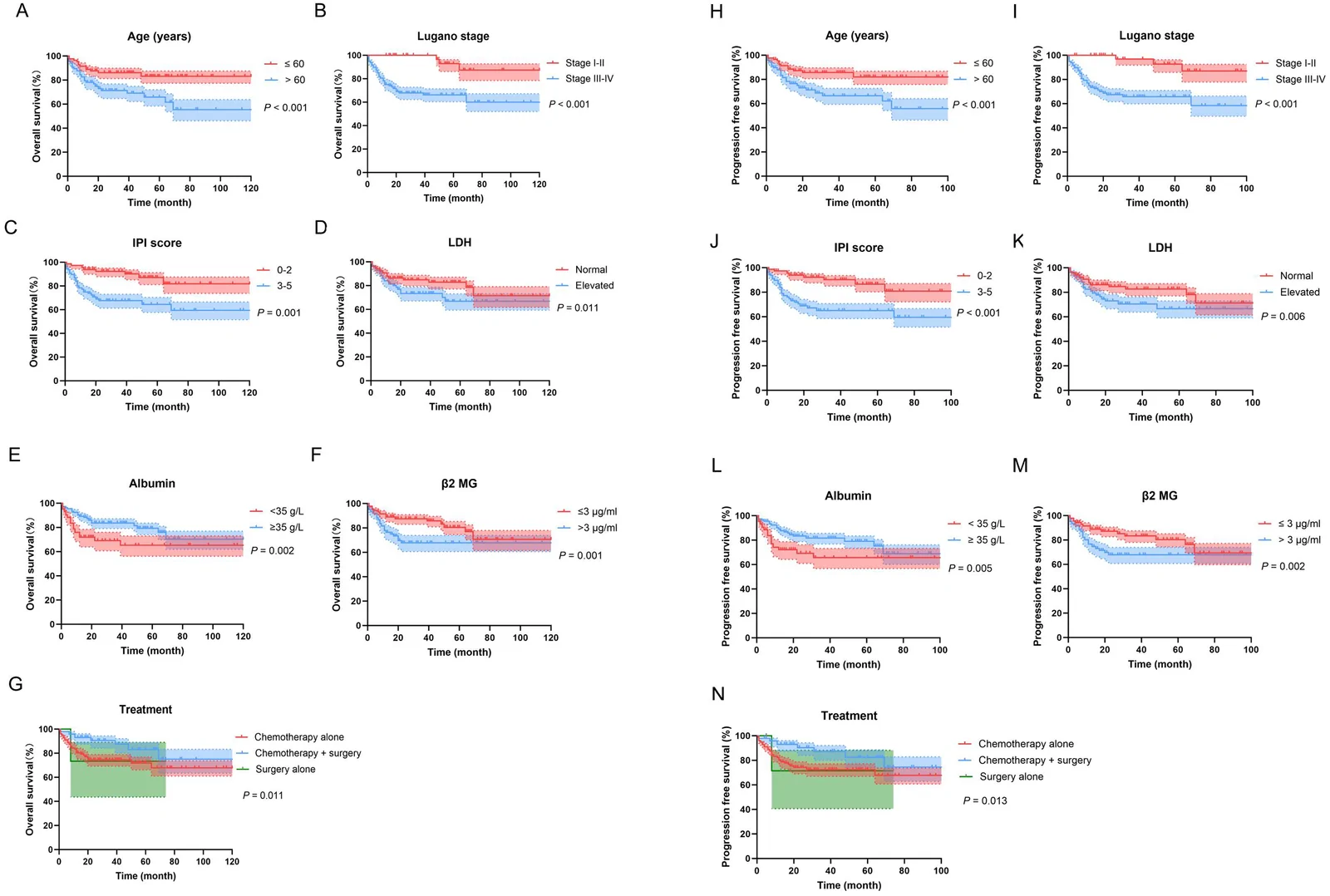
Kaplan-Meier overall survival (OS) and progression free survival (PFS) curves for prognostic factors in SI-DLBCL patients. **(A)** The OS curves of age of SI-DLBCL patients. **(B)** The OS curves of Lugano stage of SI-DLBCL patients. **(C)** The OS curves of IPI score of SI-DLBCL patients. **(D)** The OS curves of LDH of SI-DLBCL patients. **(E)** The OS curves of albumin of SI-DLBCL patients. **(F)** The OS curves of β2 Microglobulin of SI-DLBCL patients. **(G)** The OS curves of different first-line treatment of SI-DLBCL patients. **(H)** The PFS curves of age of SI-DLBCL patients. **(I)** The PFS curves of Lugano stage of SI-DLBCL patients. **(J)** The PFS curves of IPI score of SI-DLBCL patients. **(K)** The PFS curves of LDH of SI-DLBCL patients. **(L)** The PFS curves of albumin of SI-DLBCL patients. **(M)** The PFS curves of β2 Microglobulin of SI-DLBCL patients. **(N)** The PFS curves of different first-line treatment of SI-DLBCL patients.

### Cox regression analysis

Although age, serum LDH, and disease stage are core components of the composite IPI scoring system, we analyzed these individual variables separately in regression models for two key reasons: First, the composite IPI score masks the independent prognostic weight of each single biomarker in SI-DLBCL, a rare subtype lacking subtype-specific risk stratification data. Second, subgroup survival stratification (e.g., age ≤60 vs >60, normal vs elevated LDH) provides granular clinical cutoff values that cannot be derived from the summed IPI score alone. Multivariate models were constructed with both individual IPI components and the full IPI score to distinguish whether overall risk was driven by the composite index or by discrete standalone clinical factors.

The following indicators for 483 patients with SI-DLBCL were included in the Cox regression model for univariate analysis of OS: age, gender, Eastern Cooperative Oncology Group (ECOG) performance status, Lugano stage, B symptoms, pathological subtypes, IPI score, white blood cell count, serum LDH, albumin, β2-microglobulin, Ki-67 index, lymphoma location, and first-line treatment methods. The results demonstrated that age, Lugano stage, IPI score, LDH, albumin, β2-microglobulin, and first-line treatment were significant prognostic factors for OS in patients with SI-DLBCL (**Table 4**). Variables with *P* < 0.10 in the univariate Cox regression analysis were incorporated into the multivariate analysis model. The findings revealed that age and Lugano stage were independent prognostic factors affecting OS in patients with SI-DLBCL (**Table 5**). Similarly, univariate Cox regression analysis for PFS identified age, Lugano stage, IPI score, LDH, albumin, β2-microglobulin, and first-line treatment as significant prognostic factors (**Table 6**). Consistent with the OS analysis, variables with P less than 0.10 in the univariate PFS analysis were entered into the multivariate Cox regression model. After adjustment, age and Lugano stage remained the only independent prognostic factors influencing PFS in patients with SI-DLBCL (**Table 7**). Mixed-effects Cox models with center as a random intercept yielded an ICC <0.02, and age and Lugano stage remained independent prognostic factors.

**Table 4:** Univariate analysis of OS in SI-DLBCL patients with COX proportional hazards.

Index	*P* value	HR	95% CI	
			Lower limit	Upper limit
Age (> 60)	**< 0.001**	2.242	1.592	3.444
Gender	0.114	1.344	0.932	1.937
ECOG score	0.504	1.137	0.780	1.659
Lugano stage	**< 0.001**	5.731	3.146	10.443
B symptoms	0.452	1.161	0.787	1.714
Hans pathological classification	0.604	1.105	0.758	1.611
IPI score	**< 0.001**	2.965	1.933	4.547
White blood cell count	0.559	1.132	0.747	1.716
Serum LDH	**0.025**	1.527	1.055	2.210
Albumin	**0.002**	1.828	1.258	2.655
β2 microglobulin	**0.004**	1.709	1.185	2.463
Ki-67	0.657	1.100	0.722	1.675
Lymphoma site	0.245	1.332	0.822	2.157
First-line treatment	**0.009**	0.504	0.320	0.793

Statistically significant values of test statistics and P values are marked in bold black font, namely P < 0.05.

**Table 5:** Multivariate analysis of OS in SI-DLBCL patients with COX proportional hazards.

Index	*P* value	HR	95% CI	
			Lower limit	Upper limit
Age (> 60)	**0.005**	1.024	1.007	1.041
Lugano stage	**< 0.001**	4.767	2.536	8.960
IPI score	0.183	1.411	0.850	2.341
Serum LDH	0.302	1.001	1.000	1.001
Albumin	0.533	0.986	0.972	1.001
β2 microglobulin	0.419	0.986	0.899	1.023
First-line treatment	0.079	0.740	0.457	1.197

Statistically significant values of test statistics and P values are marked in bold black font, namely P < 0.05.

**Table 6:** Univariate analysis of PFS in SI-DLBCL patients with COX proportional hazards.

Index	*P* value	HR	95% CI	
			Lower limit	Upper limit
Age (> 60)	**< 0.001**	2.395	1.611	3.561
Gender	0.154	1.311	0.904	1.901
ECOG score	0.577	1.115	0.761	1.633
Lugano stage	**< 0.001**	5.491	3.011	10.012
B symptoms	0.181	1.315	0.881	1.963
Hans pathological classification	0.552	1.122	0.767	1.642
IPI score	**< 0.001**	3.362	2.149	5.259
White blood cell count	0.953	1.013	0.657	1.562
Serum LDH	**0.015**	1.589	1.094	2.309
Albumin	**0.002**	1.810	1.242	2.636
β2 microglobulin	**0.007**	1.661	1.148	2.404
Ki-67	0.487	1.162	0.762	1.772
Lymphoma site	0.665	1.121	0.669	1.878
First-line treatment	**0.008**	0.496	0.315	0.781

Statistically significant values of test statistics and P values are marked in bold black font, namely P < 0.05.

**Table 7:** Multivariate analysis of PFS in SI-DLBCL patients with COX proportional hazards.

Index	*P* value	HR	95% CI	
			Lower limit	Upper limit
Age (> 60)	**0.006**	1.024	1.007	1.041
Lugano stage	**< 0.001**	4.681	2.489	8.805
IPI score	0.143	1.455	0.881	2.403
Serum LDH	0.112	1.001	0.999	1.003
Albumin	0.977	1.000	0.986	1.014
β2 microglobulin	0.828	1.015	0.890	1.156
First-line treatment	0.096	0.741	0.456	1.203

Statistically significant values of test statistics and P values are marked in bold black font, namely P < 0.05.

### Risk factor analysis for mortality of patients with SI-DLBCL

As presented in **Table 8**, all potential prognostic factors were included in the univariate logistic regression analysis, results demonstrated that age, Lugano stage, IPI score, serum LDH, albumin, β2-microglobulin, and first-line treatment were significantly associated with mortality in patients with SI-DLBCL (all *P* < 0.05). Besides, as shown in **Figure 4**, factors with *P* < 0.05 in the univariate analysis were included in multivariate analysis, revealing that Age, Lugano stage were independent risk factors for mortality.

**Table 8:** Univariate analysis of risk factors for death in SI-DLBCL patients.

Causes of death and related factors	B	SE	Wald	df	P	Exp (B)	95.0% CI for Exp(B)	
							Lower	Upper
Age	1.083	0.224	23.488	1	**0.000**	2.955	1.907	4.580
Gender	0.404	0.216	3.504	1	0.061	1.498	0.981	2.286
ECOG score	-0.044	0.219	0.040	1	0.842	0.957	0.623	1.471
Lugano stage	1.902	0.323	34.773	1	**0.000**	6.699	3.560	12.605
B symptoms	0.130	0.226	0.334	1	0.563	1.139	0.732	1.774
Hans pathological classification	0.283	0.219	1.665	1	0.197	1.327	0.864	2.039
IPI score	1.336	0.241	30.754	1	**0.000**	3.806	2.373	6.103
White blood cell count	0.086	0.245	0.123	1	0.726	1.090	0.675	1.760
Serum LDH	0.496	0.216	5.285	1	**0.022**	1.642	1.076	2.505
Albumin	0.584	0.222	6.910	1	**0.009**	1.794	1.160	2.774
β2 microglobulin	0.668	0.215	9.629	1	**0.002**	1.950	1.279	2.974
Ki-67	-0.061	0.245	0.062	1	0.804	0.941	0.582	1.521
Lymphoma site	-0.014	0.282	0.002	1	0.961	0.986	0.568	1.713
First-line treatment	0.580	0.243	5.717	1	**0.017**	1.786	1.110	2.874

Statistically significant values of test statistics and P values are marked in bold black font, namely P < 0.05.

**Figure 4:**
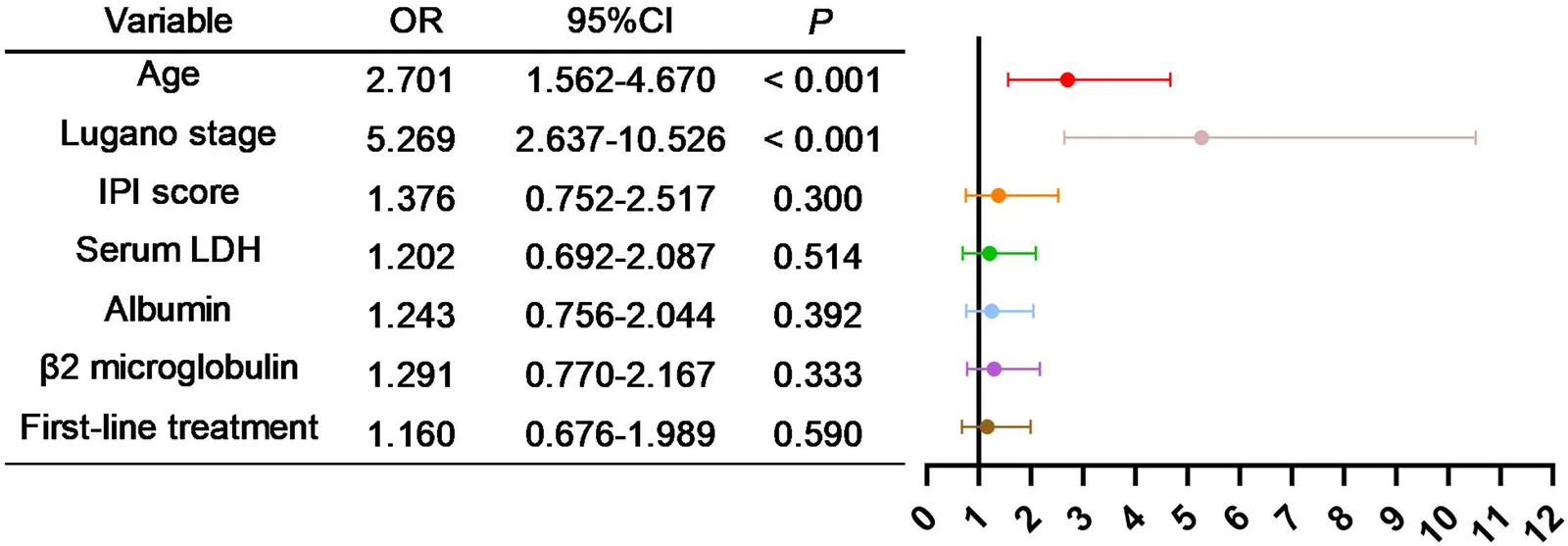
Multivariate logistic regression analysis of risk factors for death in SI-DLBCL patients.

## Discussion

Extranodal non-Hodgkin lymphoma (NHL) frequently affects the gastrointestinal tract (12). DLBCL, the most common type of NHL, accounts for 30%–40% of all malignant lymphomas (13). DLBCL predominantly affects males, with a male-to-female ratio of approximately 1.7:1. The ileum is the most commonly affected site (40%), and over 90% of lesions are localized (14). Although research on primary intestinal diffuse large B-cell lymphoma (PI-DLBCL) is increasing, little is known about secondary intestinal diffuse large B-cell lymphoma (SI-DLBCL). This study retrospectively analyzed the clinical data of 483 patients with SI-DLBCL to identify prognostic-related factors, representing one of its key innovations.

It is undeniable that SI-DLBCL is a special type of extranodal lymphoma, and its pathogenic mechanism remains elusive, however, existing literature suggests that Epstein-Barr virus (EBV) infection plays an important role (15). Chronic inflammation may lead to the overactivity and proliferation of B lymphocytes, resulting in autonomous, monoclonal expansion. Immune system dysfunction may impair apoptotic cell clearance (16). DLBCL is characterized by diffuse infiltration of medium to large cells with prominent nucleoli and abundant cytoplasm, which destroys the architecture of affected lymph nodes. DLBCL cells typically express CD19, CD20, CD22, CD45, and CD79a. Other positive markers include Bcl-6, Bcl-2, and CD10, with approximately 10%–15% of patients expressing CD30 (17). Gut-associated lymphoid tissue (GALT), predominantly located in the ileocolon region, can generate specific immune responses. Repeated antigen stimulation of GALT cells may lead to monoclonal proliferation and eventual lymphoma development (18). Immunohistochemistry (IHC) represents a cornerstone assay for detecting neoplastic B-cell populations, with diagnostic markers categorized into core and optional panels, the essential IHC biomarkers encompass CD3, CD20, Ki-67, CD10, Bcl-6, MUM-1, Bcl-2, c-myc and EBV *in-situ* hybridization (ISH), whereas supplementary markers include CD79a, CD30, CD23, CD138, and ALK, while notably, ALK immunohistochemical staining carries diagnostic significance for specific DLBCL subgroups, as elaborated in a recent study that highlighted the practical utility of ALK-targeted IHC within diagnostic workflows for this lymphoma entity (19). Interestingly, one study (20) reported marked copy number gains at the ZCCHC7/PAX5 locus in transformed lymphomas or double-hit lymphomas. Moreover, PAX5-TSS2 mutations have been identified in multiple DLBCL cell lines and primary human DLBCL specimens. Deletion or point mutation of PAX5-TSS2 strengthens the interaction between the PAX5 enhancer region and the ZCCHC7 promoter, which in turn upregulates ZCCHC7 mRNA expression. Accordingly, mutations at the PAX5 promoter in lymphoma cells facilitate ZCCHC7 expression. Collectively, these findings suggest that the ZCCHC7/PAX5 locus may serve as a promising therapeutic target and diagnostic molecular biomarker for DLBCL.

From the perspective of clinical baseline data, the results of our study indicate that male patients with SI-DLBCL are more prevalent (61.70%), with a median age at onset of 60.5 years (range: 18–84 years), consistent with previous reports (6). Early clinical manifestations lack specificity and may include abdominal pain, low back pain, fatigue, fever, vomiting, and weight loss. Additionally, the clinical features of SI-DLBCL may be masked by the primary tumor’s manifestations (21). In this study, only 182 cases (37.68%) presented with abdominal pain as their chief complaint, underscoring the difficulty of early diagnosis. Differential diagnoses include intestinal ulcers, intestinal obstruction, and colorectal cancer (22). Despite the rising incidence of lymphoma, intestinal lesions should still be considered in clinical practice to avoid misdiagnosis.

Common imaging diagnostic methods for SI-DLBCL include ultrasound, contrast-enhanced imaging, computed tomography (CT), positron emission tomography-computed tomography (PET-CT), and magnetic resonance imaging (MRI), as well as endoscopic examinations such as gastroscopy, colonoscopy, and capsule endoscopy. PET-CT is highly recommended for lymphoma patients due to its ability to precisely locate enteral and extraintestinal lesions through 18F-fluorodeoxyglucose (18F-FDG) metabolism (23). Endoscopy is crucial for diagnosing intestinal lymphoma, enabling visual inspection of the diseased intestine, lesion localization, and immediate biopsy. It improves preoperative diagnostic accuracy and allows for visual comparison of lesions before and after treatment, offering less trauma and lower costs compared to surgery (24, 25). Routine initial examinations include blood, urine, and stool tests, biochemistry, coagulation profiles, and D-dimer levels, though results may not always be abnormal. Histopathological biopsy remains the gold standard for diagnosis, with repeated biopsies recommended if clinically suspected but initially negative.

Treatment allocation across participating centers uniformly followed international and domestic DLBCL guidelines published between 2015 and 2025. Surgical intervention was only administered to patients presenting with acute intestinal complications or unresectable bulky local lesions, while R-CHOP and variant rituximab-containing regimens were standardized as first-line systemic therapy for all other SI-DLBCL patients. The findings of this study indicate that the first-line treatment plan predominantly involved chemotherapy. Specifically, 322 cases (66.67%) received chemotherapy alone, while 146 cases (30.23%) underwent a combination of chemotherapy and surgical intervention. For patients with SI-DLBCL, non-surgical treatment regimens in clinical practice encompass chemotherapy, radiotherapy, and immunotherapy, among others. The standard first-line chemotherapy regimen is R-CHOP (rituximab combined with cyclophosphamide, epirubicin, vindesine, and dexamethasone) (26). In addition to conventional standard chemotherapy regimens, various targeted drugs are available for selection. CD20 is a 33–37 kDa non-glycosylated protein expressed on the surface of normal and malignant B lymphocytes. The phosphorylation level of CD20 in malignant proliferating B lymphocytes exceeds that in normal B lymphocytes. Phosphorylated and activated CD20 functions as a calcium channel, participating in the activation of B lymphocytes. Consequently, targeting CD20 can mediate changes in signaling pathway expression, thereby inhibiting the proliferation of malignant B lymphocytes (27). Since the 1990s, CD20 monoclonal antibodies (e.g., rituximab) have significantly advanced the treatment of B-cell malignancies. Rituximab has been applied to follicular lymphoma (FL) and DLBCL. It also plays a role in the standard treatment regimens for DLBCL and chronic lymphocytic leukemia (CLL) (28). However, resistance to rituximab in some relapsed/refractory patients and certain adverse reactions to the drug have prompted researchers worldwide to develop new alternative therapies. Currently, several novel CD20 monoclonal antibodies include obutuzumab, ofatumab, and ubutuximab (29).

It should be noted that the observed survival advantage in the chemotherapy-plus-surgery group in univariate analysis may be largely attributable to confounding by indication rather than a true therapeutic benefit. In our cohort, surgical intervention was selectively reserved for patients with acute intestinal complications (perforation, massive bleeding, complete bowel obstruction) or bulky localized lesions, as detailed in the Methods section. These patients represent a clinically distinct subgroup with different baseline characteristics and disease biology compared to those treated with chemotherapy alone. Indeed, after adjusting for confounding variables in multivariate Cox regression analysis, first-line treatment modality was no longer an independent prognostic factor for OS (*P* = 0.079) or PFS (*P* = 0.096). Therefore, the apparent benefit of combined surgery and chemotherapy should be interpreted with caution and cannot be generalized as a recommendation for upfront surgical debulking in all patients. Prospective, randomized controlled trials or well-designed propensity-score matched studies are warranted to definitively address this question.

It is noteworthy that the B-cell receptor (BCR) signaling pathway plays a critical role in the proliferation, differentiation, and development of B cells. Bruton’s tyrosine kinase (BTK) inhibitors effectively block BCR signal transduction, thereby inhibiting the proliferation, migration, and survival of malignant B lymphocytes. Current marketed BTK inhibitors are effective in treating FL, CLL, marginal zone lymphoma (MZL), Waldenström macroglobulinemia (WM), and also demonstrate efficacy in DLBCL treatment (30–32). Relevant literature reports (33) that in early-stage disease (stages I-II), involved site radiation therapy (ISRT) could be administered following a short course (three cycles) of R-CHOP treatment or treated with a full course (six cycles) of R-CHOP. For advanced-stage disease (stages III-IV), six cycles of R-CHOP could be used for treatment. Radiotherapy remains an effective treatment modality. Studies have demonstrated that consolidation radiotherapy correlates with improved OS and PFS (34, 35). Prospective data from 134 patients with inpatient or aggressive intestinal lymphoma revealed a tumor control rate of 100% after radiotherapy ± chemotherapy (36). If SI-DLBCL arises due to immunotherapy, studies suggest that immunotherapy for the primary disease should be suspended, followed by chemotherapy or radiotherapy for SI-DLBCL. Some studies indicate that tumor necrosis factor antagonists (TNFA), such as infliximab, adalimumab, and etanercept, may also be effective in treating SI-DLBCL (37).

SI-DLBCL could lead to persistent malabsorption in patients, resulting in suboptimal clinical outcomes. Patients with SI-DLBCL who underwent surgery for intestinal obstruction or perforation during the early stages exhibited longer OS without increased postoperative morbidity or mortality. However, perforation was identified as a key factor associated with poor prognosis (38). Chemotherapy for small intestinal NHL can cause perforation, which poses a life-threatening risk. Approximately 9% of patients with gastrointestinal lymphoma experience perforation, with the risk being over six times higher in aggressive lymphomas compared to indolent B-cell lymphomas. Perforation is primarily caused by transmural damage induced by tumors or tissue necrosis following chemotherapy (39). A 37-year retrospective study of patients with gastrointestinal lymphoma found that 9% developed perforations, with 55% occurring post-chemotherapy. The most common sites of perforation were the small intestine (59%), followed by the large intestine (22%) and stomach (16%) (40). EBV positivity, older age, and perforation correlate with lower survival rates, whereas the expression of programmed cell death ligand 1 (PD-L1) on microenvironmental immune cells is associated with better prognoses (41). This study demonstrates that age and Lugano stage are independent prognostic factors influencing OS and PFS in patients with SI-DLBCL. Currently, there are relatively few reports on the prognostic factors of SI-DLBCL (42), and our novel findings in this study would provide a direction for further research.

The innovation of this study lies in the multi-center retrospective clinical investigation of patients with SI-DLBCL conducted in collaboration with tertiary hospitals across different regions of China. This approach breaks away from previous studies that were limited to single-center case reports or retrospective analyses, which were relatively rare in clinical practice. The strengths of our research include the integration of multi-center clinical data, comprehensive data analysis, systematic investigation into the clinical characteristics of SI-DLBCL, and an evaluation of current diagnostic and therapeutic strategies. Additionally, we identified prognostic risk factors and assessed the applicability of existing treatment regimens. These contributions are expected to facilitate earlier diagnosis, optimize treatment decision-making, and potentially improve patient outcomes.

However, this study bears several limitations. First, incomplete molecular subtyping data (GCB/non-GCB, MYC/BCL2 double-hit status and gene rearrangements) in many patients, arising from inconsistent historical pathological testing across centers, precluded molecular subgroup prognostic analysis. Second, treatment protocols evolved over the 11-year enrollment period, leading to temporal therapeutic heterogeneity. Third, few patients received novel targeted or CAR-T therapies, limiting reliable subgroup comparisons. Future prospective multicenter studies with unified centralized molecular testing are needed to verify our results and optimize risk stratification for SI-DLBCL. Fourth, as a retrospective non-randomized cohort, first-line treatment allocation was not randomized. Surgical intervention was selectively reserved for patients with acute intestinal complications (perforation, massive bleeding, obstruction) or bulky localized disease, introducing confounding by indication; therefore, the apparent survival advantage of the chemotherapy-plus-surgery arm in univariate analysis should not be interpreted as a causal treatment benefit, and the optimal role of surgery awaits validation in propensity-score matched or prospective randomized studies. These limitations suggest that future research should focus on expanding the number of collaborating medical centers and examining the impact of emerging diagnostic and therapeutic approaches on the prognosis and clinical outcomes of SI-DLBCL patients.

## Conclusion

In summary, patients with SI-DLBCL predominantly consist of middle-aged and elderly men. Early clinical manifestations lack specificity, necessitating pathology as the gold standard for diagnosis, supplemented by imaging and laboratory tests to assess lesion extent and systemic metastasis. Chemotherapy-based immunochemotherapy remains the primary first-line treatment modality for SI-DLBCL. Surgical intervention is selectively indicated for patients with acute intestinal complications or bulky localized lesions, and its impact on long-term survival requires further prospective validation. Age and Lugano stage are independent prognostic factors affecting OS and PFS in patients with SI-DLBCL. Future research will involve collaboration with more multiple centers to explore the molecular pathogenic mechanisms, clinical characteristics and diagnostic/treatment experiences of rare-site extranodal lymphomas, providing additional references for hematology.

## Data Availability

The original contributions presented in the study are included in the article/supplementary material. Further inquiries can be directed to the corresponding author.
